# Prognostic value of sarcopenia in aortic valve replacement: a systematic review and meta-analysis

**DOI:** 10.3389/fnut.2025.1529270

**Published:** 2025-07-29

**Authors:** Jie He

**Affiliations:** ^1^School of Clinical Medicine, Chengdu Medical College, Chengdu, Sichuan, China; ^2^Department of Pulmonary and Critical Care Medicine, The First Affiliated Hospital of Chengdu Medical College, Chengdu, Sichuan, China

**Keywords:** SAVR, TAVR, sarcopenia, prevalence, systematic review, meta-analysis

## Abstract

**Objective:**

This study aimed to quantify the prevalence of sarcopenia in patients undergoing surgical aortic valve replacement (SAVR) or transcatheter aortic valve replacement (TAVR), and to assess its association with mortality risk.

**Methods:**

Relevant studies were identified through searches of the PubMed, Cochrane Library, Excerpta Medica Database (Embase), Web of Science, and China National Knowledge Infrastructure (CNKI) from inception through July 1, 2025. The prevalence of sarcopenia and its 95% confidence interval (CI) were calculated, with heterogeneity evaluated using the *I*^2^ statistic. The link between sarcopenia and mortality following SAVR/TAVR was quantified by hazard ratio (HR) or odds ratio (OR) with 95% CI. Statistical analyses were conducted using Stata 11.0.

**Results:**

Thirty-eight studies were included, with 6 focusing on patients undergoing SAVR and 32 on those undergoing TAVR. Sarcopenia was defined by skeletal mass index in 16 studies, while only 2 studies adopted criteria combining reduced muscle mass with low muscle strength and/or reduced physical performance. Sarcopenia’s prevalence among SAVR patients was 31.3% (95% CI 25.3–37.6%). In this cohort, sarcopenia was linked to a significantly higher risk of long-term (≥1 year) mortality (HR = 3.10, 95% CI 2.00–4.79, *p* < 0.001). In contrast, the prevalence of sarcopenia in TAVR patients was 43.7% (95% CI 38.6–48.9%), with sarcopenia also correlating with increased long-term (>2 year) mortality (HR = 1.25, 95% CI 1.09–1.44, *p* = 0.001). These associations remained consistent across various follow-up durations, definitions of sarcopenia, and ethnic groups.

**Conclusion:**

Despite the variation in diagnostic criteria, sarcopenia is significantly prevalent in both SAVR and TAVR populations, with a clear association with elevated long-term mortality following these procedures.

**Systematic review registration:**

https://www.crd.york.ac.uk/PROSPERO/, identifier CRD42024606633.

## 1 Introduction

Given advancements in extracorporeal circulation and cardiac surgical techniques, surgical aortic valve replacement (SAVR) has become the predominant cardiac intervention for elderly patients with aortic valve disease (1). While transcatheter aortic valve replacement (TAVR) now offers an alternative approach for treating aortic stenosis, SAVR remains advantageous for patients experiencing aortic regurgitation, infective endocarditis, ascending aortic aneurysms, or other valvular disorders (2, 3). Optimal treatment for elderly patients, however, necessitates a thorough evaluation of benefits and risks, with accurate postoperative mortality assessment remaining a fundamental concern for clinicians.

Baseline functional status is recognized as a strong indicator of surgical risk (4). Identifying specific patient risk factors is essential in guiding clinical decisions on whether TAVR or SAVR represents the optimal approach or if aortic valve replacement (AVR) is potentially non-beneficial. Modifiable factors among these may highlight possible avenues for additional interventions accompanying valve replacement to enhance post-procedural outcomes. Sarcopenia, a condition with implications for adverse clinical results in SAVR/TAVR, is characterized by age-associated reductions in skeletal muscle mass, strength, and physical function (5). Globally, sarcopenia prevalence spans 8–36% in individuals under 60 and 10–27% in those aged 60 or older (6). Prior research has consistently linked sarcopenia with cardiovascular diseases such as coronary heart disease, heart failure, and aortic stenosis (7, 8), positioning it as a risk factor for cardiovascular disease progression and negatively influencing patient prognosis (9, 10). Notably, sarcopenia independently predicts mortality in cardiac surgery patients and is prevalent among elderly patients, where it correlates with poorer post-surgical outcomes (11). Consequently, comprehensive sarcopenia evaluation is increasingly viewed by researchers as integral to refining decision-making in SAVR/TAVR.

As the population ages, the prevalence of age-related frailty increases sharply, which increases the risk of poor health status of older adults (12). Frailty syndrome is becoming increasingly prevalent with population aging, characterized by diminished physiological reserve and impaired stress response capacity. Research indicates that this state of heightened vulnerability exerts significant adverse effects on elderly patients undergoing AVR, manifesting as worsened postoperative dependency, increased readmission rates, elevated mortality, and other negative clinical outcomes (13). Frailty holds significant importance in assessing the risks of TAVR, particularly given the advanced age of many patients (14). However, frailty assessments are often subjective and rely on indirect measurements. Sarcopenia, one of the key biological contributors to frailty (15), can be utilized to evaluate surgical risks in TAVR patients. In the pursuit of objective frailty parameters, previous studies have extensively investigated the analysis of body composition metrics, with a particular focus on muscle mass and muscle strength (16). These parameters are widely applied in cardiac risk assessment. Numerous studies have examined sarcopenia’s predictive role in outcomes post-SAVR/TAVR, yet findings remain inconsistent and, in some cases, contradictory. For instance, Brouessard et al. (17) observed no significant association between sarcopenia and one-year rehospitalization or mortality rates following TAVR. Conversely, Heidari et al. (18) identified sarcopenia as an independent predictor of mortality in TAVR patients. Additionally, Mirzai et al. (19) demonstrated that unilateral pectoralis muscle measurements from preoperative cardiac magnetic resonance imaging may serve as a supplementary metric to conventional risk scores in predicting mortality risk post-SAVR. Despite these varied findings, no meta-analysis has yet synthesized evidence on sarcopenia’s impact on clinical outcomes in SAVR/TAVR patients. This study was therefore undertaken to (i) quantify sarcopenia prevalence in SAVR/TAVR patients, and (ii) assess sarcopenia’s association with post-SAVR/TAVR mortality.

## 2 Materials and methods

### 2.1 Searching strategy and methods

The study adhered to the updated Preferred Reporting Items for Systematic Reviews and Meta-Analyses (PRISMA) (2020) guidelines, with the protocol registered in PROSPERO database (CRD42024606633).^1^ PubMed, Cochrane Library, Excerpta Medica Database (Embase), Web of Science, and China National Knowledge Infrastructure (CNKI) were searched, covering records from inception to July 1, 2025, with no language restrictions to ensure a thorough capture of relevant studies. Two researchers executed this search, utilizing keywords encompassing but not limited to: “TAVI” OR “TAVR” OR “transcatheter aortic valve implantation” OR “transcatheter aortic valve replacement”; “aortic valve replacement” OR “AVR” OR “surgical aortic valve replacement” OR “SAVR”; AND “sarcopenia” OR “sarcopenic” OR “muscle mass” OR “muscle strength” OR “hand strength” OR “grip strength” OR “muscle atrophy” OR “muscle wasting.” Related references in these studies were achieved. The search strategy employed across databases has been documented in detail (Supplementary Table 1).

### 2.2 Inclusion and exclusion criteria

Eligible studies were selected: (i) Participants: patients with sarcopenia undergoing SAVR/TAVR; (ii) Exposure: sarcopenia, defined per each study’s criteria due to the lack of a standardized definition; (iii) Comparison: patients without sarcopenia; (iv) Outcome: association between sarcopenia and mortality risk; (v) Study design: prospective or retrospective cohort studies. Exclusion criteria: (i) absence of definitely reported diagnostic criteria for sarcopenia, rendering data extraction infeasible; (ii) narrative reviews, comments, editorials, case series, conference abstracts, or related letters; (iii) studies treating sarcopenia as an outcome rather than a prognostic factor; (iv) publications in non-English languages.

### 2.3 Data extraction

Data extraction from each study was executed in a standardized form. Two reviewers independently gathered the following details: first author’s name, publication year, study design, location, patient sample size, mean or median age, body mass index (BMI), Society of Thoracic Surgeons (STS) score, sarcopenia definitions, muscle mass and function metrics, sarcopenia prevalence, and outcomes. For studies lacking specific data, attempts were made to contact the authors for supplementary information or clarification; studies were excluded if responses were not obtained.

### 2.4 Quality assessment of included studies

Assessment of study quality was conducted independently by at least two authors employing a standardized assessment scale. The Newcastle-Ottawa Scale (NOS) scale was applied to case–control and cohort studies, comprising criteria on study population selection (4 items, one point each), comparability (1 item, two points), and exposure or outcome (3 items, one point each); scores between 0–3 signified low quality, 4–6 moderate quality, and 7–9 high quality. Disagreements during the quality assessment were addressed through discussions by reviewers or resolved by expert arbitration when necessary.

### 2.5 Statistical analysis

This meta-analysis focused on two primary outcomes: (1) sarcopenia prevalence in SAVR /TAVR patients and (2) the association between sarcopenia and mortality. A single-proportion meta-analysis was used to pool the prevalence estimates, with subgroup analyses stratified by sarcopenia definition method and sex. The prevalence of sarcopenia was used as the common effect size in this meta-analysis. The effect of sarcopenia on mortality was quantified through pooled hazard ratio (HR) or odds ratio (OR) with 95% confidence interval (CI), applying a random- or fixed-effects model based on study variance. The heterogeneity test employed *I*^2^ and Cochran’s Q statistics, with significant heterogeneity indicated by a *p*-value ≤ 0.1 or *I*^2^ ≥ 50% (20). Predefined subgroup analyses examined variables such as follow-up duration, diagnostic criteria, ethnicity, and study design. Sensitivity analysis Sensitivity analyses will be conducted, excluding studies from the analysis one by one (21). Given the limited studies per outcome, meta-regression analysis was not conducted to examine sources of heterogeneity. Begg’s funnel plots were conducted to evaluate the publication bias qualitatively. A (Begg’s) funnel plot was used to visually detect the presence of publication bias in the meta-analysis. Begg’s test and Egger’s test were performed to quantitatively assess the publication bias (22). Statistical significance was defined as a two-tailed *p* < 0.05, with all analyses performed in STATA 11.0.

## 3 Results

### 3.1 Literature screening process

**Figure 1:**
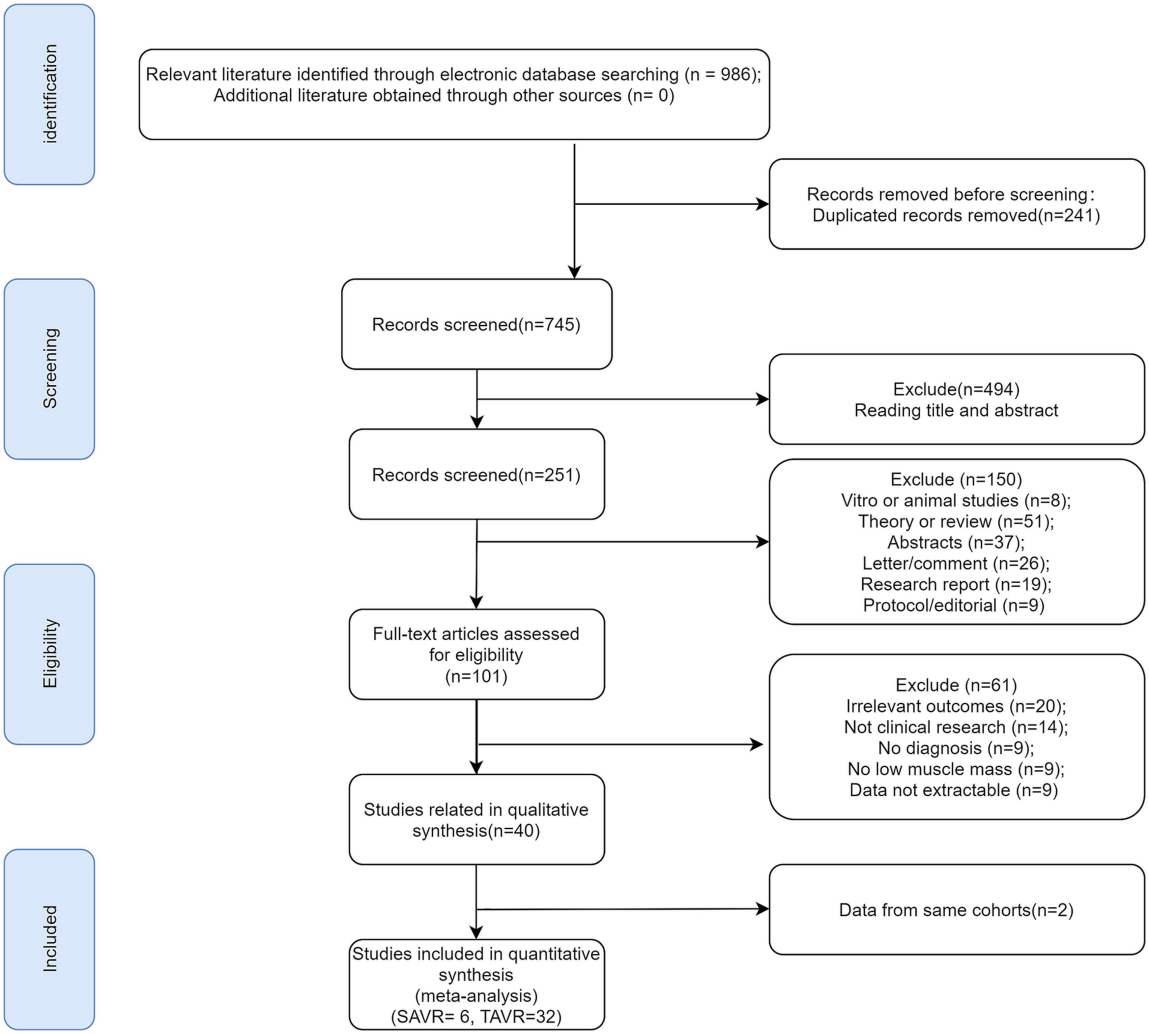
Flow diagram of literature screening.

A total of 986 articles were initially retrieved through the search (strategy detailed in Supplementary Table 1). Following duplicate removal, 745 unique articles remained, from which titles and abstracts were reviewed, yielding 251 for further analysis. Of these, 101 full texts underwent screening, and 61 articles were excluded for reasons specified in Figure 1. After additional exclusions of partially irrelevant or duplicate studies (*n* = 2), 38 eligible articles were included. In SAVR patients, one article (11) analyzed ORs, 2 examined HRs (11, 19), 6 focused solely on prevalence (11, 19, 23–26). For TAVR patients, 31 articles addressed prevalence (16–18, 23, 26–52), 19 examined HRs (16–18, 27, 29, 32, 34, 36, 37, 39–41, 44, 45, 47, 49, 51–53), 5 focused solely on ORs (18, 33, 36, 45, 46). The identification process was illustrated in the flowchart in Figure 1.

### 3.2 Literature characteristics

Table 1 provides an overview of study characteristics included in this study. SAVR patients: Six studies contributed to the qualitative analysis, with all studies published post-2016. Among these, six studies used retrospective designs. Sample sizes ranged from 15 to 874, with patient ages averaging between 67 and 85 years. The participant population covered diverse regions: three studies in Asia, and three in America. TAVR patients: Similarly, 32 studies were analyzed qualitatively, all published after 2016, comprising twenty-two retrospective and 10 prospective cohort studies. Sample sizes varied between 19 and 1,375, with a mean age range of 67 to 85 years. NOS scores indicated moderate to high study quality. Table 1 also details the diagnostic criteria for sarcopenia commonly applied. Twenty-eight studies identified sarcopenia via diminished muscle mass measured by CT scan, one used ultrasound, and another employed the sarcopenia index. Two additional studies (49, 50) utilized low muscle strength (grip strength) and physical performance (gait speed) as sarcopenia indicators. This finding highlighted the limited use of comprehensive sarcopenia criteria (only 2 studies using combined muscle mass and strength/performance) is valuable and underscores a gap in standardization.

**Table 1:** Study characteristics.

Study, year	Country	Study design	Study type	Mean/median age (years)	BMI (kg/m^2^)	STS score	Assessment of sarcopenia	Definition of sarcopenia	Prevalence of sarcopenia (*n*/*N*)		Study quality	Main outcomes
van Erck et al. (2024) (27)	Netherlands	Retrospective	CS	80.0 ± 7.0	27.1 ± 5.0	NA	CT: L3-SMD	L3-SMD <10.7 HU in men, <1.2 HU in women	400	1,199	7	B, D
Stein et al. (2024) (16)	USA	Prospective	CS	82.9 (76.8–87.4)	27.3 (24.1–31.6)	3.66 (2.53–5.39)	CT: L4-PMAi	L4-PMAi <7.5 cm^2^/m^2^ in men, <5.2 cm^2^/m^2^ in women	184	445	8	B, D
Solla-Suarez et al. (2024) (35) (Canada)	Spain	Prospective	CS	84.8 ± 5.3	24.5 ± 3.5	NA	CT: L4-PMA	L4-PMA <22 cm^2^ in men, <12 cm^2^ in women	126	605	8	B, D
Solla-Suarez et al. (2024) (37) (Spain)	Spain	Prospective	CS	83.4 ± 4.3	29.8 ± 4.4	NA	Grip strength; Vscan extend handheld ultrasound system (Total muscle thickness)	Grip strength <27 kg in men, <16 kg in women; Total muscle thickness ≥28.3 mm	42	150	7	B, D
Persits et al. (2024) (40)	USA	Retrospective	CS	80.6 ± 7.7	28.7 ± 5.8	NA	CT: T9-T12-SMI	T9-T12-SMI <266.5 cm^2^/m^2^ in men, <182.1 cm^2^/m^2^ in women	42	184	7	B, D
Pekař et al. (2024) (41)	Czech Republic	Retrospective	CS	79.7 (74.9–83.3)	28.9 (25.9–32.6)	NA	CT: L3-SMI	L3-SMI <42.44 cm^2^/m^2^	322	866	5	B, D
Demirel et al. (2024) (38)	Switzerland	Retrospective	CS	82.8 ± 5.95	25.6 (22.7–29.7)	NA	CT: L3-nTMA	NA	250	500	7	B
Pesarini et al. (2023) (42)	Italy	Prospective	CS	81.6 ± 4.6	25.6	3.37	CT: L3-SMI	L3-SMI <55.4 cm^2^/m^2^ in men, <38.9 cm^2^/m^2^ in women	56	99	6	B
Imamura et al. (2023) (29)	Japan	Retrospective	CS	85.0 (83.0–88.0)	21.7 (19.4–24.4)	4.60 (3.9–6.1)	CT: L3-PMAi	L3-PMAi <10.7 × 10 cm^3^/m^2^	152	322	7	B, D
Hecht et al. (2022) (30)	Austria	Retrospective	CS	83.03 ± 4.68	25.80 ± 4.19	3.19 ± 1.87	CT: L3-PMAi	L3-PMAi ≤757.16 mm^2^/m^2^ in men, ≤569.88 mm^2^/m^2^ in women	59	179	8	B
Fukuda et al. (2022) (23)	Japan	Prospective	CCS	84.8 ± 3.6	NA	NA	SMI, Grip strength, gait speed	Grip strength <18 kg or walking speed <1.0 m/s, and SMI < 5.7 kg/m^2^ only in females	16	19	5	A, B
Van de Velde-Van De Ginste et al. (2021) (49)	Belgium	Retrospective	CS	84.9 ± 5.3	25.7 ± 4.5	5.0 ± 3.1	Gait speed and grip strength	Gait speed <0.8 m/s and Grip strength <27 kg in men, <16 kg in women	24	125	8	B, D
Uchida et al. (2021) (31)	Japan	Prospective	CS	85.0 ± 5.0	22.5 ± 3.3	6.0 ± 4.1	PMAi	NA	35	71	7	B
Romeo et al. (2021) (39)	Argentina	Retrospective	CS	83.9 ± 5.7	27.2 ± 4.4	3.3 ± 1.7	Sarcopenia index	SI ≤66	33	100	8	B, D
Tzeng et al. (2020) (28)	Taiwan	Retrospective	CS	79.2 ± 7.8	23.9 ± 3.9	11.03 ± 3.9	CT: L3-SMD	10.4 HU	73	182	8	B
van Mourik et al. (2019) (32)	Netherlands	Retrospective	CS	82.6	27.1	4.6	CT: L3-PMAi	9.1 × 10 mm^2^/m^2^	192	576	7	B, D
Garg et al. (2017) (33)	USA	Retrospective	CS	83.3 ± 6.5	28.1 ± 5.6	6.9 ± 3	CT: L3-PMAi	L3-PMA <4.15 cm^2^/m^2^ in men, <3.47 cm^2^/m^2^ in women	76	152	5	B
Paknikar et al. (2016) (26)	USA	Retrospective	CS	80.0 ± 8.9	29.6 ± 7.1	6.5 ± 4.5	CT: L4-TPA	NA	40	124	7	A, B
Gallone et al. (2022) (36)	Italy	Retrospective	CS	81.0 ± 6.0	NA	4.4 ± 3.6	CT: L3-SMI, L3-PMA	L3-SMI <55.4 cm^2^/m^2^ in men, <38.9 cm^2^/m^2^ in women; L3-PMA <20.3 cm^2^ in men, <11.8 cm^2^ in women	SMI: 192; PMA: 117	391	8	B, D
Hsu et al. (2021) (50)	Taiwan	Prospective	CCS	78.16 ± 7.95	23.66 ± 3.75	NA	Calf circumference, grip strength, and gait speed	Handgrip strength <27 kg in men, <16 kg in women, Gait speed ≤0.8 m/s, Calf circumference <34 cm in men, <33 cm in women	47	81	8	B
Brouessard et al. (2021) (17)	France	Retrospective	CS	84	26.4	NA	CT: L3-SMI; Gait speed	L3-SMI <55.4 cm^2^/m^2^ in men, <38.9 cm^2^/m^2^ in women; Gait speed measurement ≤0.8 m/s	56	182	7	B, D
Brown et al. (2022) (43)	Canada	Retrospective	CS	80.7 ± 9.6	29.2 ± 13.8	NA	CT: L3-SMI	L3-SMI <52.6 cm^2^/m^2^ in men, <40.9 cm^2^/m^2^ in women	116	468	9	B
Yoon et al. (2021) (44)	Korea	Prospective	CS	78.9 ± 5.2	NA	4.1 ± 3.0	CT: L3-SMI	L3-SMI ≤38.9 cm^2^/m^2^ in women, ≤31.3 cm^2^/m^2^ in men	174	522	7	B, D
Tokuda et al. (2020) (45)	Japan	Retrospective	CS	NA	NA	NA	CT: L3-SMI	L3-SMI <55.4 cm^2^/m^2^ in men, <38.9 cm^2^/m^2^ in women	802	1,375	7	B, D
Krishnan et al. (2019) (34)	USA	Retrospective	CS	82.0 ± 8.0	27.9 ± 10.0	7.1 ± 5.5	CT: L3-SMI, L4-PMAi	L3-SMI <50.0 cm^2^/m^2^ in men, <35.0 cm^2^/m^2^ in women; L4-PMAi <12.0 cm^2^/m^2^ in men, <8.0 cm^2^/m^2^ in women	151	381	8	B, D
Heidari et al. (2019) (18)	USA	Retrospective	CS	80.9 ± 8.9	NA	NA	CT: L4-SMI	L3-SMI <55.4 cm^2^/m^2^ in men, <38.9 cm^2^/m^2^ in women	374	602	8	B, D
Nemec et al. (2017) (46)	USA	Retrospective	CS	82.3 ± 10.0	27.3 ± 6.1	7.1 ± 5.3	CT: L3, Th7 and Th12-SMI	L3-SMI <55.4 cm^2^/m^2^ in men, <38.9 cm^2^/m^2^ in women; Th7-SMI <46.5 cm^2^/m^2^ in men, <32.3 cm^2^/m^2^ in women; Th12-SMI <42.6 cm^2^/m^2^ in men, <30.6 cm^2^/m^2^ in women	53	157	7	B
Mok et al. (2016) (47)	Canada	Prospective	CS	80.0 ± 8.0	26.8 ± 5.8	6.9 ± 3.9	CT: L3-SMI	L3-SMI <55.4 cm^2^/m^2^ in men, <38.9 cm^2^/m^2^ in women	293	460	8	B, D
Dahya et al. (2016) (46)	USA	Prospective	CS	81.0 ± 10.0	28.0 ± 5.0	NA	CT: L3-SMI	L3-SMI <55.0 cm^2^/m^2^ in men, <39.0 cm^2^/m^2^ in women	73	104	7	B
Mirzai et al. (2023) (19)	USA	Retrospective	CS	67 ± 9	27.1 ± 4.8	6.7 (5.5–12.2)	CT: pectoralis SMI	Pectoralis SMI < 9.16 cm^2^/m^2^ in men, <6.21 cm^2^/m^2^ in women	45	133	8	B, C
Lee et al. (2022) (11)	Korea	Retrospective	CS	72 (68–75)	24.2 (21.9–26.3)	2.2 (1.6–3.1)	CT: L3-SMI	L3-SMI < 55.0 cm^2^/m^2^ in men, <39.0 cm^2^/m^2^ in women	292	874	7	A, C
Kondo et al. (2022) (24)	Japan	Retrospective	CS	78.1 ± 5.2	22.5 ± 3.8	NA	CT: L4-PMAi	L4-PMAi <956 mm^2^/m^2^ in men, <730 mm^2^/m^2^ in women	29	140	5	A
Hawkins et al. (2018) (25)	USA	Retrospective	CS	81 (77–85)	NA	NA	CT: L4-PMAi	NA	60	240	5	A

BMI, body mass index; STS, Society of Thoracic Surgeons Predicted Risk of Mortality score; L3, 3rd lumbar vertebra; L4, 4th lumbar vertebra; T9, the ninth thoracic vertebra; T12, the twelfth thoracic vertebra; SMI, skeletal muscle index; SMD, skeletal muscle density; PMA, psoas muscle area; PMAi, indexed PMA; CS, cohort study; CCS, case–control study; NA, not applicable; A, prevalence of sarcopenia in surgical aortic valve replacement; B, prevalence of sarcopenia in transcatheter aortic valve replacement; C, mortality risk in surgical aortic valve replacement; D, mortality risk in transcatheter aortic valve replacement.

### 3.3 SAVR

#### 3.3.1 Prevalence of sarcopenia

**Figure 2:**
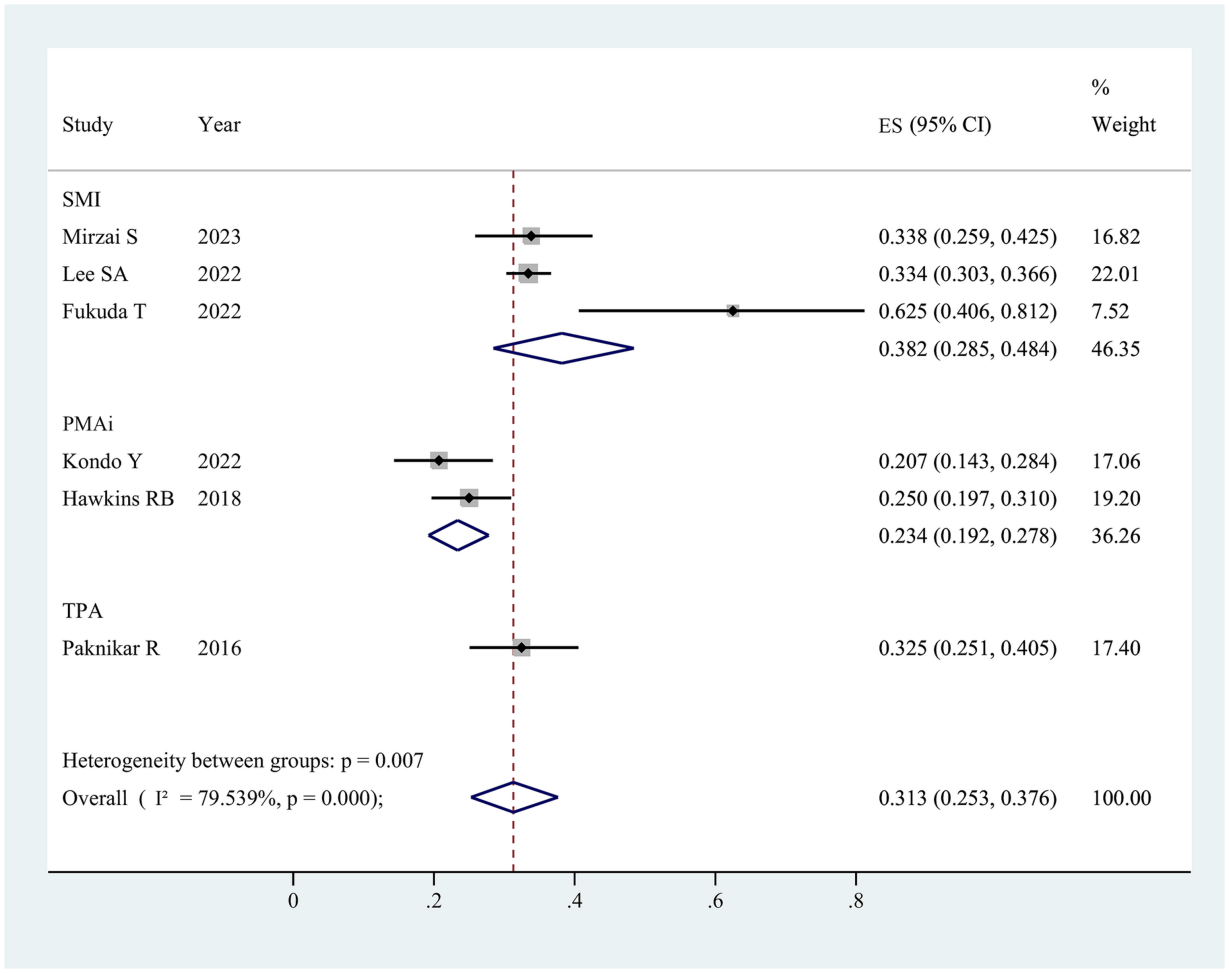
The pooled overall prevalence of sarcopenia in patients undergoing surgical aortic valve replacement. ES, effect size; 95%CI, 95% confidence interval; SMI, skeletal muscle index; PMAi, psoas muscle area index; TPA, total psoas area.

In the six studies analyzed, sarcopenia prevalence varied between 20.7 and 62.5% (Table 1), with an overall pooled prevalence of 31.3% (95% CI 25.3–37.6%) (Figure 2). Subgroup analysis based on differing definitions of sarcopenia revealed substantial variation across groups. When defined by skeletal mass index (SMI), the prevalence was 38.2% (95% CI 28.5–48.4%, three studies); for psoas muscle area index (PMAi), the prevalence was 23.4% (95% CI 19.2–27.8%, two studies); and for TPA, a single study reported a prevalence of 32.5% (95% CI 25.1–40.5%).

##### 3.3.1.1 Publication bias and sensitivity analysis

Funnel plots, along with Egger’s and Begg’s tests, assessed potential selection bias in the literature. The funnel plot demonstrated symmetry (Supplementary Figure 1A), and both Egger’s test (*p* = 0.707) and Begg’s test (*p* = 0.828) indicated no significant publication bias. Sensitivity analysis, conducted by sequential exclusion of individual studies, revealed no statistically significant variation in outcomes, confirming the stability of the results (Supplementary Figure 1B).

#### 3.3.2 Sarcopenia and mortality risk

##### 3.3.2.1 HRs

Two studies (11, 19) examined the correlation between sarcopenia and long-term mortality (≥1 year) post-SAVR, presenting multivariate analysis results that identified sarcopenia as a significant predictor of elevated mortality risk, with a pooled adjusted HR of 3.10 (95% CI 2.00–4.79, *p* < 0.001; Supplementary Figure 2). Additionally, the included studies demonstrated minimal heterogeneity (*I*^2^ = 0%, *p* = 0.46).

##### 3.3.2.2 ORs

A study (11) identified an association between sarcopenia and 30-day post-TAVR mortality. According to Lee SA, sarcopenia correlated with an increased short-term mortality risk, with an OR of 2.46 (95% CI: 1.63–3.79, *p* < 0.05).

### 3.4 TAVR

#### 3.4.1 Prevalence of sarcopenia

**Figure 3:**
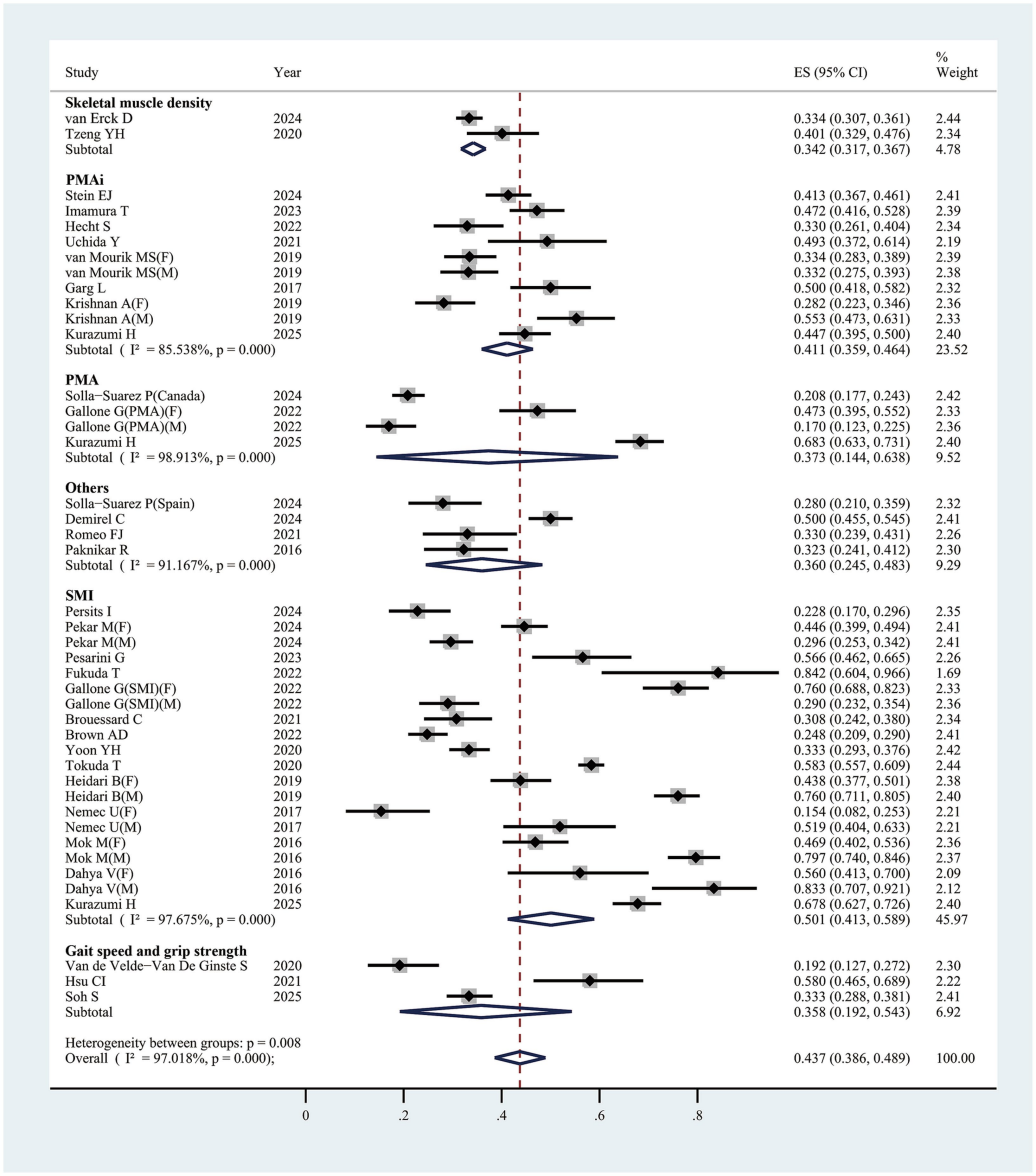
The pooled overall prevalence of sarcopenia in patients undergoing transcatheter aortic valve replacement. ES, effect size; 95%CI, 95% confidence interval; SMI, skeletal muscle index; PMAi, psoas muscle area index; PMA, psoas muscle area.

In the 31 studies reviewed, sarcopenia prevalence varied from 15.4 to 83.3% (Table 1), with a pooled estimate of 43.7% (95% CI, 38.6–48.9%) (Figure 3). Subgroup analysis by differing sarcopenia definitions revealed substantial variations across groups. Specifically, using SMI as the criterion, prevalence reached 50.1% (95% CI, 40.3–58.9%, 20 studies); with psoas muscle area index (PMAi), prevalence was 41.1% (95% CI, 35.9–46.4%, ten studies); psoas muscle area (PMA) indicated a prevalence of 37.3% (95% CI, 14.4–63.8%, four studies); gait speed and grip strength showed a prevalence of 33.4% (95% CI, 27.0–40.0%, two studies) (Table 2). Among male patients, the prevalence was 50.5% (95% CI, 33.4–67.5%) compared to 46.2% (95% CI, 36.4–56.1%) in females, indicating a slightly higher incidence in males (Supplementary Figure 3) (Table 2). The results of subgroup analyses are shown in Table 2.

**Table 2:** Subgroup analyses on the incidence of sarcopenia in different conditions.

Subgroup analysis (*n*)	ES (95% CI)	*p*-value	*I*^2^ (%)	*P*_h_
Overall (43)	0.437 (0.386, 0.489)	<0.001	97.01%	<0.001
Assessment of sarcopenia				
SMI (20)	0.501 (0.413, 0.589)	<0.001	97.68%	<0.001
PMAi (10)	0.411 (0.359, 0.464)	<0.001	85.54%	<0.001
PMA (4)	0.373 (0.144, 0.638)	<0.001	98.91%	0.016
SMD (2)	0.342 (0.317, 0.367)	<0.001	97.20%	<0.001
Gait speed and grip (2)	0.334 (0.270, 0.400)	<0.001	66.91%	<0.001
Others (5)	0.355 (0.267, 0.448)	<0.001	90.50%	<0.001
Gender				
Male (9)	0.505 (0.334, 0.675)	<0.001	98.34%	<0.001
Female (10)	0.462 (0.364, 0.561)	<0.001	94.44%	<0.001
Mixed (24)	0.403 (0.343, 0.465)	<0.001	96.92%	<0.001
Study design				
Prospective (17)	0.497 (0.399, 0.595)	<0.001	97.20%	<0.001
Retrospective (26)	0.402 (0.342, 0.464)	<0.001	97.00%	<0.001
Ethnicity				
Caucasian (31)	0.411 (0.351, 0.473)	<0.001	96.87%	<0.001
Asian (11)	0.520 (0.436, 0.607)	<0.001	96.22%	<0.001
Latino (1)	0.330 (0.239, 0.431)	<0.001	NA	NA

SMI, skeletal muscle index; PMAi, psoas muscle area index; PMA, psoas muscle area; SMD, skeletal muscle density; *P*_h_, *P*_heterogeneity_; NA, not applicable; ES, effect size.

##### 3.4.1.1 Publication bias and sensitivity analysis

Potential publication bias in the literature selection was assessed through funnel plots, Egger’s test, and Begg’s test. Symmetry observed in the funnel plot (Supplementary Figure 4A) suggested minimal bias. Neither Egger’s test (*p* = 0.507 > 0.05) nor Begg’s test (*p* = 0.121 > 0.05) yielded statistically significant results, confirming an absence of notable publication bias. After sequentially excluding individual studies, sensitivity analysis revealed no statistically significant impact on outcomes, affirming the stability of the results (Supplementary Figure 4B).

#### 3.4.2 Sarcopenia and mortality risk

##### 3.4.2.1 HRs

**Figure 4:**
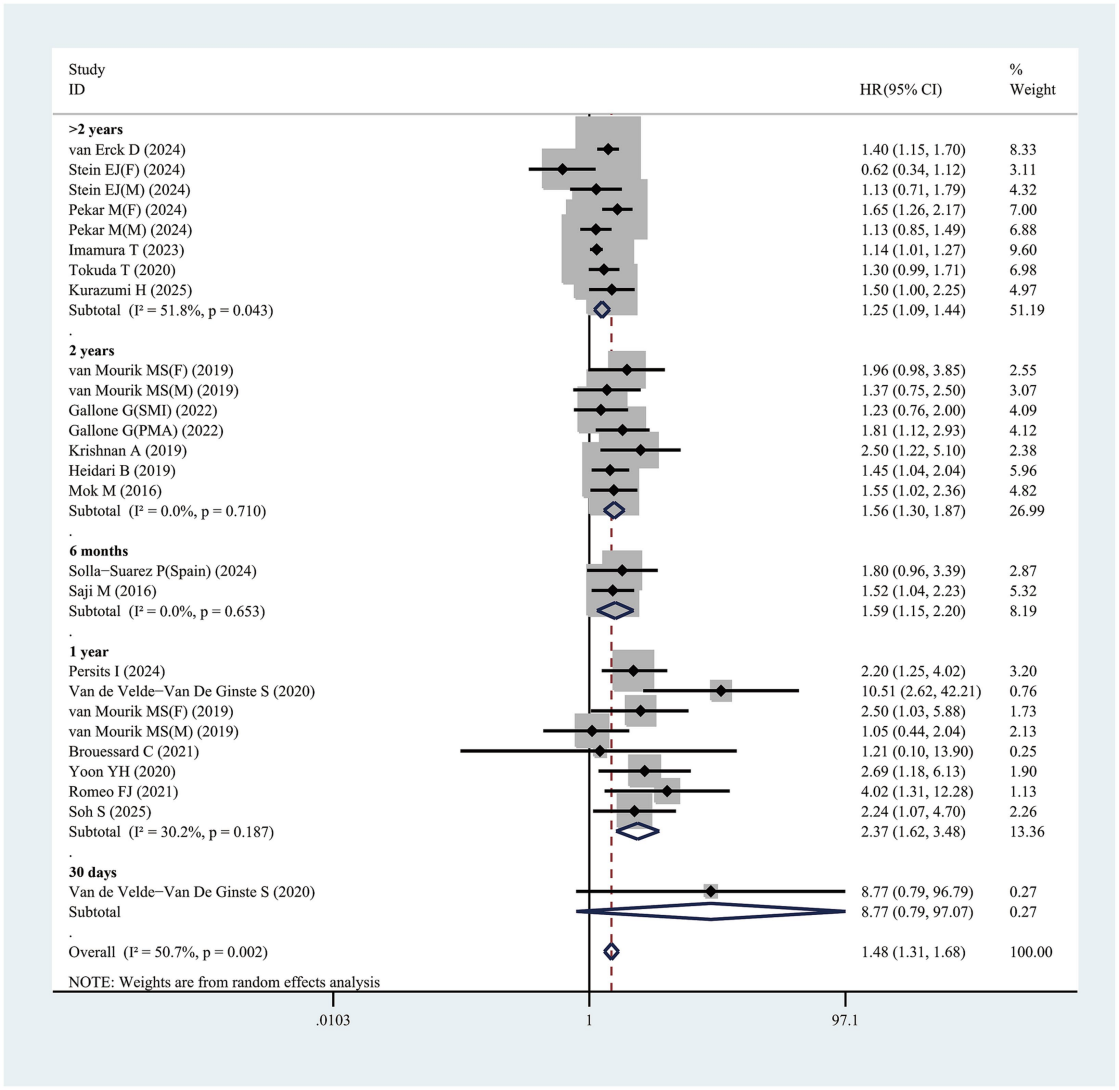
Forest plot for the association between sarcopenia and the risk of mortality after transcatheter aortic valve replacement (pooled HR value). HR, hazard ratio; 95%CI, 95% confidence interval.

Nineteen studies evaluated the link of sarcopenia to mortality risk following TAVR. All studies provided multivariable analysis, demonstrating a significant correlation between sarcopenia and increased mortality risk, with a pooled adjusted HR of 1.48 (95% CI 1.31–1.68, *p* < 0.001; Figure 4). Substantial heterogeneity was observed across studies (*I*^2^ = 50.7%, *p* = 0.001). Publication bias test used funnel plots, Egger’s, and Begg’s test (Supplementary Figure 5A). After excluding 1 study at a time, sensitivity analysis showed no significant change in results, affirming the finding robustness (Supplementary Figure 5B).

Furthermore, this study identified that follow-up duration influenced mortality risk in sarcopenic patients, with longer follow-up times correlating with higher risk. For patients with follow-up exceeding 2 years, sarcopenia was linked to a HR of 1.25 (95% CI 1.09–1.44, *p* = 0.001) (Figure 4). In those followed up for exactly 2 years, the mortality risk associated with sarcopenia rose further, with HR of 1.56 (95% CI 1.30–1.87, *p* < 0.001) (Figure 4). Patients with a 1-year follow-up exhibited a HR of 2.37 (95% CI 1.62–3.48, *p* < 0.001), while for a 6-month follow-up, the HR was 1.59 (95% CI 1.15–2.20, *p* = 0.005) (Figure 4). One study reported a 30-day follow-up duration. However, Van de Velde-Van De Ginste et al. (49) reported no significant association between sarcopenia and mortality risk in TAVR patients (HR = 8.77, 95% CI 0.79–97.07, *p* = 0.077).

In addition, this study revealed that among the analyzed subgroups, variations in sarcopenia definitions (e.g., SMI or alternative criteria) may affect the association between sarcopenia and mortality risk. Patients with sarcopenia diagnosed through SMI exhibited a significantly elevated mortality risk, with HR of 1.43 (95% CI 1.25–1.65, *p* < 0.001) (Table 3). Furthermore, sarcopenia identified via the PMAi method showed statistically significant correlation with mortality risk, reflected in a HR of 1.31 (95% CI 1.04–1.64, *p* = 0.022), as detailed in Table 3. For patients diagnosed with sarcopenia using other diagnostic approaches (e.g., ultrasound, gait speed, grip strength, sarcopenia index, standardized PMA, PMA, muscle density), the condition remained significantly linked to increased mortality risk, yielding a HR of 1.92 (95% CI 1.42–2.60, *p* < 0.001) (Table 3). Notably, subgroup analysis by sex indicated no significant association between sarcopenia and mortality risk for either male (HR = 1.15, 95% CI = 0.93–1.42, *p* = 0.205) or female patients (HR = 1.45, 95% CI = 0.85–2.48, *p* = 0.176) (Table 3). Additional subgroup analysis results were presented in Table 3.

**Table 3:** Subgroup analysis of the adjusted HR in patients with TAVR.

Subgroup analysis (*n*)	ES (95% CI)	*p*-value	*I*^2^ (%)	*P*_h_
Overall (26)	1.48 (1.31,1.68)	<0.001	50.7%	0.002
Assessment of sarcopenia				
SMI (9)	1.43 (1.25, 1.65)	<0.001	13.6%	0.321
PMAi (9)	1.31 (1.04, 1.64)	0.022	49.0%	0.047
Others (8)	1.92 (1.42, 2.60)	<0.001	52.1%	0.041
Gender				
Male (4)	1.15 (0.93, 1.42)	0.205	0.0%	0.939
Female (4)	1.45 (0.85, 2.48)	0.176	72.3%	0.013
Mixed (18)	1.59 (1.36, 1.85)	<0.001	53.4%	0.004
Study design				
Prospective (5)	1.29 (0.86, 1.94)	0.227	63.9%	0.026
Retrospective (21)	1.52 (1.33, 1.74)	<0.001	49.3%	0.006
Ethnicity				
Caucasian (21)	1.52 (1.31, 1.76)	<0.001	43.3%	0.019
Asian (5)	1.39 (1.10, 1.75)	0.005	53.6%	0.097
Follow up time				
30 days (1)	8.77 (0.79, 96.79)	0.077	NA	NA
6 months (2)	1.59 (1.15, 2.20)	0.005	0.0%	0.653
1 year (8)	2.37 (1.62, 3.48)	<0.001	30.2%	0.187
2 years (7)	1.56 (1.30, 1.87)	<0.001	0.0%	0.710
>2 years (8)	1.25 (1.09, 1.44)	0.001	51.8%	0.043

SMI, skeletal muscle index; PMAi, psoas muscle area index; *P*_h_, *P*_heterogeneity_; NA, not applicable; TAVR, transcatheter aortic valve replacement; HR, hazard ratio.

##### 3.4.2.2 ORs

**Figure 5:**
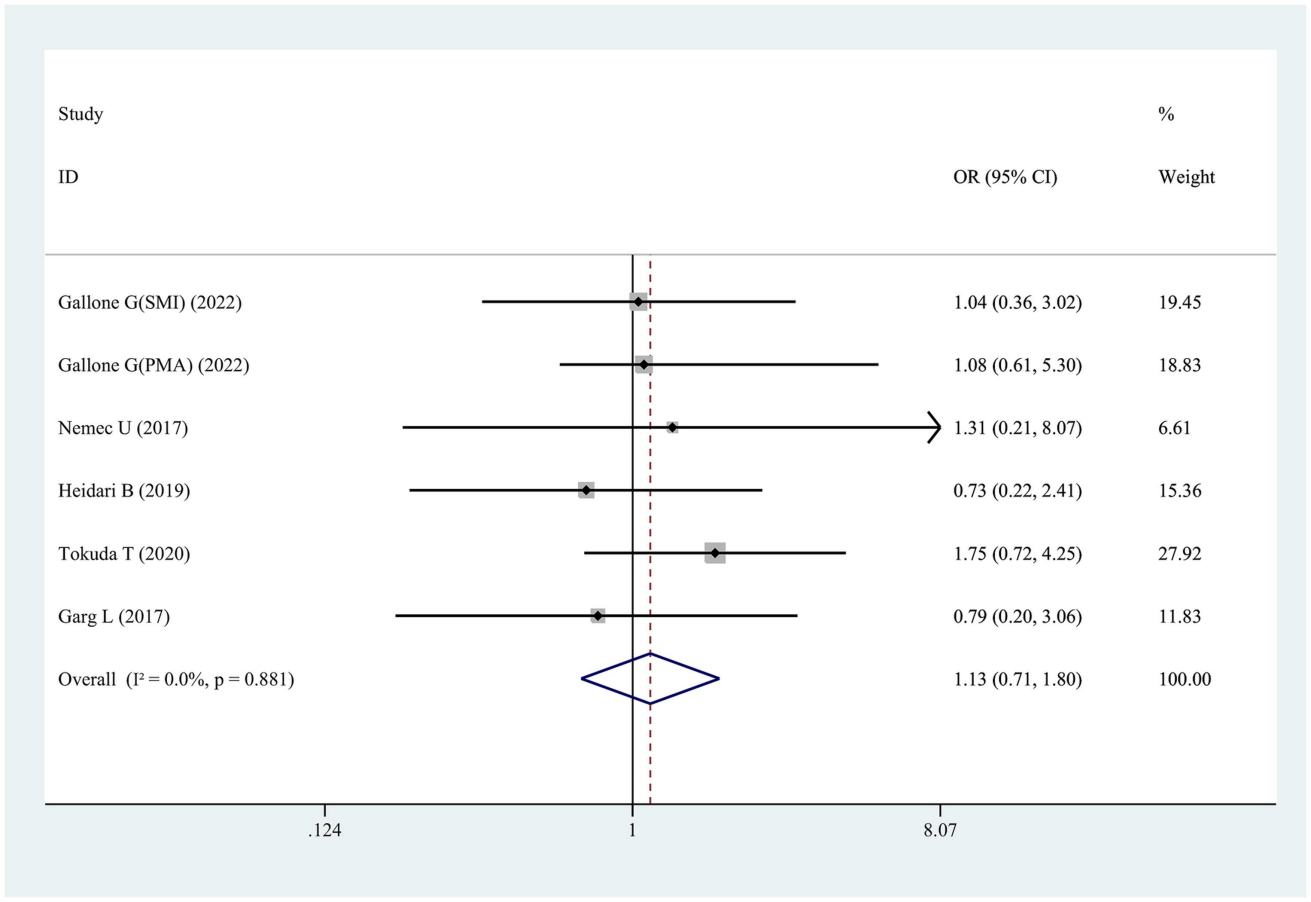
Forest plot for the association between sarcopenia and the risk of short-term mortality after transcatheter aortic valve replacement (pooled OR value). OR, odds ratio; 95%CI, 95% confidence interval.

Six studies examined the link of sarcopenia to short-term mortality (≤30 days) following TAVR (Figure 5). No statistically significant association was found, with ORs spanning 0.73 to 1.75 and a pooled OR of 1.13 (95% CI 0.71–1.80, *p* = 0.616; Figure 5).

## 4 Discussion

This study presents the first comprehensive systematic review and meta-analysis evaluating sarcopenia prevalence among patients with aortic stenosis (SAVR/TAVR). Sarcopenia is notably prevalent and increases with advancing age, yet it remains underdiagnosed and its clinical significance is often undervalued (54). Notably, patients selected for TAVR—representing a high-risk, elderly demographic with severe aortic stenosis ineligible for SAVR—exhibit a higher incidence of sarcopenia compared to SAVR patients. Clinicians are therefore encouraged to assess sarcopenia severity in both patient cohorts, as preoperative sarcopenia screening can enhance management planning and improve perioperative and postoperative risk assessment. Furthermore, this analysis explored the association between sarcopenia and patient outcomes across these surgical approaches, suggesting that sarcopenia may influence long-term postoperative mortality risk, potentially linked to frailty and malnutrition. Variability in diagnostic criteria likely contributed to differing sarcopenia prevalence rates across studies, but no significant disparity in sarcopenia prevalence was observed between the SAVR and TAVR groups. Additionally, race, sex, and study design were not found to impact sarcopenia prevalence.

Sarcopenia was initially defined as age-related skeletal muscle mass loss. More recently, it has been broadly characterized by both diminished muscle mass and reduced muscle strength (e.g., weak grip strength) and/or physical performance (e.g., slow gait speed) (55). In cardiovascular research, however, sarcopenia is predominantly diagnosed based on skeletal muscle mass, typically assessed by measuring muscle area at L3 vertebra using CT imaging (56). This method was employed by most studies included in the current review. A few studies, however, utilized chest CT scans at the thoracic vertebra levels T7 and T12 for muscle mass estimation. Given that CT scans in patients undergoing TAVR or SAVR are generally performed on the chest rather than the abdomen (57), the link to skeletal muscle measurements at L3 and T12 becomes particularly relevant, with stronger associations observed between these two levels (46). Further investigation is needed to establish the optimal threshold for defining sarcopenia using chest CT scans in TAVR and SAVR patients.

The prevalence of sarcopenia exhibited significant variability across studies in this meta-analysis, largely due to inconsistent cutoffs for low muscle mass. While cutoff values for sarcopenia are sex-specific, prevalence tends to be higher among male patients than female patients undergoing TAVR/SAVR, indicating a potential influence of sex on sarcopenia rates in this population. Prior research also suggests a stronger link between sarcopenia and functional decline in men compared to women (58, 59), possibly due to the higher incidence of smoking among men. Cigarette smoke, rich in free radicals, induces oxidative stress in skeletal muscle (60), and prolonged smoking contributes to neuromuscular junction degeneration (61). In this meta-analysis, smoking status likely served as a confounding factor in the sex-based differences in sarcopenia prevalence; however, subgroup analyses by smoking status were not feasible due to a lack of sex-specific smoking data in the included studies. Future research should further investigate the impact of sex differences on sarcopenia.

In noncardiac procedures, such as elective spine surgery, sarcopenia independently predicts intensive care needs and postoperative transfusion requirements (62). In cases of acute mesenteric ischemia, sarcopenic patients experience reduced postoperative complications and lower 30-day mortality rates (63). Defined by psoas muscle mass, sarcopenia also serves as an independent predictor of 2-year mortality, major complications, and complication severity following major colorectal surgery (64). As an objective indicator of frailty, sarcopenia reliably predicts early morbidity and mortality post-spine surgery (53). In the context of cardiac surgery, sarcopenia correlates with poorer prognostic outcomes (26, 65), including prolonged hospital stays among older adults (66). Consequently, sarcopenia has been increasingly recognized as a relevant predictor of adverse outcomes in cardiac procedures. This study identified sarcopenia as a significant predictor of mortality risk following TAVR/SAVR procedures. While findings suggest a limited impact of sarcopenia on short-term mortality among these patients, this may be attributed to the limited sample size across included studies. Most studies assessed sarcopenia using the SMI method, associating it with an elevated risk of postoperative mortality. In subgroup analysis, nine studies using PMAi to diagnose sarcopenia yielded a pooled HR of 1.31, also indicating the significant association between sarcopenia and post-TAVR mortality risk. In this study, we further separated participants into subgroups for ethnicity stratified analysis. In either Asian population or Caucasian population, sarcopenia was broadly and robustly associated with a higher risk of mortality. The relationship between skeletal muscle mass and post-TAVR/SAVR mortality likely involves the vital role of skeletal muscle in frailty syndromes (67), though underlying mechanisms remain poorly defined. Skeletal muscle serves as the primary amino acid reservoir, and depletion impairs several recovery-critical functions (68). TAVR/SAVR patients with diminished muscle mass exhibit reduced muscle protein synthesis and heightened vulnerability to deconditioning post-intervention, exacerbated by inadequate nutrition and limited physical activity (69). Emerging research indicating the anti-inflammatory and anti-apoptotic properties of skeletal muscle may offer insights into the observed outcomes (70). Patients with sarcopenia, however, may derive limited benefit from these protective effects (71). Post-operative muscle mass reduction extends beyond survival outcomes in TAVR/SAVR, as sarcopenic patients show an increased likelihood of requiring transfer to rehabilitation facilities (33). Consequently, CT has become a valuable tool in assessing muscle mass, facilitating early identification of sarcopenic patients who may benefit from targeted interventions—such as protein supplementation and physical rehabilitation—to improve frailty status post-TAVR/SAVR. With advances in medicine, nanoparticles may hold promise for the diagnosis and treatment of sarcopenia (72). Furthermore, several studies have shown positive impact of physical therapy and nutritional interventions on sarcopenia (73, 74), suggesting that preoperative strategies, including exercise training and dietary supplementation, may enhance outcomes for patients undergoing TAVR.

In addition, sarcopenia demonstrated a strong association with elevated long-term mortality following TAVR/SAVR, aligning with evidence from patients undergoing other cardiac procedures, including heart valve surgery (75), PCI (76), and endovascular aneurysm repair (77). Additionally, the studies analyzed indicated that sarcopenia served as an independent predictor of 1-year mortality risk, even after adjustments for STS scores and relevant covariates, reinforcing its value as a practical and accessible alternative to conventional risk scores in assessing TAVR/SAVR patients.

Analysis of studies included in this review indicated no significant link between sarcopenia and short-term (30-day) mortality in TAVR patients. This observation supports the view that short-term post-TAVR outcomes remain favorable and unaffected by sarcopenia, suggesting TAVR’s safety across a wide patient demographic with varying degrees of muscle mass decline during short-term follow-up. However, this result contrasts with findings from other cardiac surgeries. For instance, Ganapathi et al. reported the association of frailty with discharge to other than home and 30-day mortality in proximal aortic surgery patients (78), while Lee et al. (11) found sarcopenia correlated with higher 30-day mortality and extended hospital stays. Given that only six studies in this meta-analysis assessed 30-day mortality, further research is needed to clarify these findings. Accordingly, future investigations should explore the relationship between sarcopenia, short-term mortality, and other adverse outcomes post-TAVR.

However, several limitations affect this meta-analysis. First, the analysis only included English-language publications, which may exclude relevant studies in other languages. Second, the studies employed varying definitions of sarcopenia, which were adopted in this review. Although most studies rely on CT-based sarcopenia assessments, discrepancies in scan locations and cutoff values across studies may have introduced bias, potentially contributing to publication bias. Notably, sex-specific cutoff values for sarcopenia have been previously derived through optimal stratification, with thresholds of 38.9 cm^2^/m^2^ (women) and 55.4 cm^2^/m^2^ (men) being widely adopted; however, cutoff values in post-SAVR/TAVR studies remain inconsistent. Finally, limited reporting on postoperative complications related to sarcopenia is due to limited studies evaluating early mortality, with such interpretations vulnerable to competing risks bias. Thus, while the observed effects of sarcopenia on early outcomes merit attention, further research is needed for this domain.

## 5 Conclusion

In summary, the study reveals a high incidence of sarcopenia among SAVR/TAVR patients, correlating with increased long-term mortality but showing no significant association with short-term mortality. While existing guidelines advocate preoperative body composition measurement and functional assessment as screening measures (79), the findings support a stronger recommendation for pre-surgical muscle mass evaluation to enhance risk stratification and outcome prediction. Given the high prevalence and prognostic importance of sarcopenia in SAVR and TAVR patients, integrating nanoformulated therapies could offer a future-oriented, precision-based strategy to improve muscle health and reduce mortality. Future research should focus on translational applications of nanomedicine in cardiac geriatrics to bridge the gap between diagnosis and therapeutic efficacy in managing sarcopenia.

## Data Availability

The original contributions presented in the study are included in the article/Supplementary material, further inquiries can be directed to the corresponding author.

## Footnotes

^1^ https://www.crd.york.ac.uk/PROSPERO/
