# Supplementary material for: Prognostic value of sarcopenia in aortic valve replacement: a systematic review and meta-analysis
Source: Front Nutr. 2025 Jul 29;12:1529270. doi: 10.3389/fnut.2025.1529270 (PMC12339327; doi:10.3389/fnut.2025.1529270)
Supplement: Supplementary file 1 [file Table_1.docx]

**Table S1. Search strategies in each database---** **July 1st, 2025**

| **Database** | **Search strategies** | **Results** |
| --- | --- | --- |
| **PubMed** | #1 "Sarcopenia"[Mesh]  #2 sarcopenia[Title/Abstract] OR sarcopenic[Title/Abstract] OR muscle mass[Title/Abstract] OR muscle strength[Title/Abstract] OR hand strength[Title/Abstract] OR grip strength[Title/Abstract] OR muscle atrophy[Title/Abstract] OR muscle wasting[Title/Abstract]  #3 #1 OR #2  #4 aortic valve replacement [Title/Abstract] OR AVR [Title/Abstract] OR surgical aortic valve replacement [Title/Abstract] OR SAVR [Title/Abstract] OR TAVI [Title/Abstract] OR TAVR [Title/Abstract] OR transcatheter aortic valve implantation[Title/Abstract] OR transcatheter aortic valve replacement  #5 #3 AND #4 | 199 |
| **Web of**  **Science** | #1 ((TS=(Sarcopenia)) OR TI=(sarcopenia OR sarcopenic OR muscle mass OR muscle strength OR hand strength OR grip strength OR muscle atrophy OR muscle wasting)) OR AB=(sarcopenia OR sarcopenic OR muscle mass OR muscle strength OR hand strength OR grip strength OR muscle atrophy OR muscle wasting)  #2 ((TS=(aortic valve replacement)) OR TI=(AVR OR surgical aortic valve replacement OR SAVR OR TAVI OR TAVR OR transcatheter aortic valve implantation OR transcatheter aortic valve replacement)) OR AB=( aortic valve replacement OR AVR OR surgical aortic valve replacement OR SAVR OR TAVI OR TAVR OR transcatheter aortic valve implantation OR transcatheter aortic valve replacement)  #3 #1 AND #2 | 290 |
| **Embase** | #1 'sarcopenia'/exp OR sarcopenia:ti,ab,kw OR sarcopenic:ti,ab,kw OR 'muscle mass':ti,ab,kw OR 'muscle strength':ti,ab,kw OR 'hand strength':ti,ab,kw OR 'grip strength':ti,ab,kw OR 'muscle atrophy':ti,ab,kw OR 'muscle wasting':ti,ab,kw  #2 ' aortic valve replacement ' OR ' AVR ':ti,ab,kw OR ' surgical aortic valve replacement ':ti,ab,kw OR ' SAVR ':ti,ab,kw OR TAVI:ti,ab,kw OR ' TAVR ':ti,ab,kw OR transcatheter aortic valve implantation:ti,ab,kw OR ' transcatheter aortic valve replacement':ti,ab,kw  #3 #1 AND #2 | 376 |
| **Cochrane library** | #1 MeSH descriptor: [Sarcopenia] explode all trees  #2 (sarcopenia OR sarcopenic OR muscle mass OR muscle strength OR hand strength OR grip strength OR muscle atrophy OR muscle wasting):ti,ab,kw (Word variations have been searched)---45,863  #3 #1 OR #2  #4 (aortic valve replacement OR AVR OR surgical aortic valve replacement OR SAVR OR TAVI OR TAVR OR transcatheter aortic valve implantation OR transcatheter aortic valve replacement):ti,ab,kw  #5 #3 AND #4 | 106 |
| **CNKI** | #1 (SU=Sarcopenia) OR (TKA=sarcopenia OR sarcopenic OR muscle mass OR muscle strength OR hand strength OR grip strength OR muscle atrophy OR muscle wasting)  #2 (SU=aortic valve replacement) OR (TKA=AVR OR surgical aortic valve replacement OR SAVR OR TAVI OR TAVR OR transcatheter aortic valve implantation OR transcatheter aortic valve replacement)  #3 #1 AND #2 | 15 |
