# Supplementary figures and images for: Prognostic value of sarcopenia in aortic valve replacement: a systematic review and meta-analysis
Source: Front Nutr. 2025 Jul 29;12:1529270. doi: 10.3389/fnut.2025.1529270 (PMC12339327; doi:10.3389/fnut.2025.1529270)

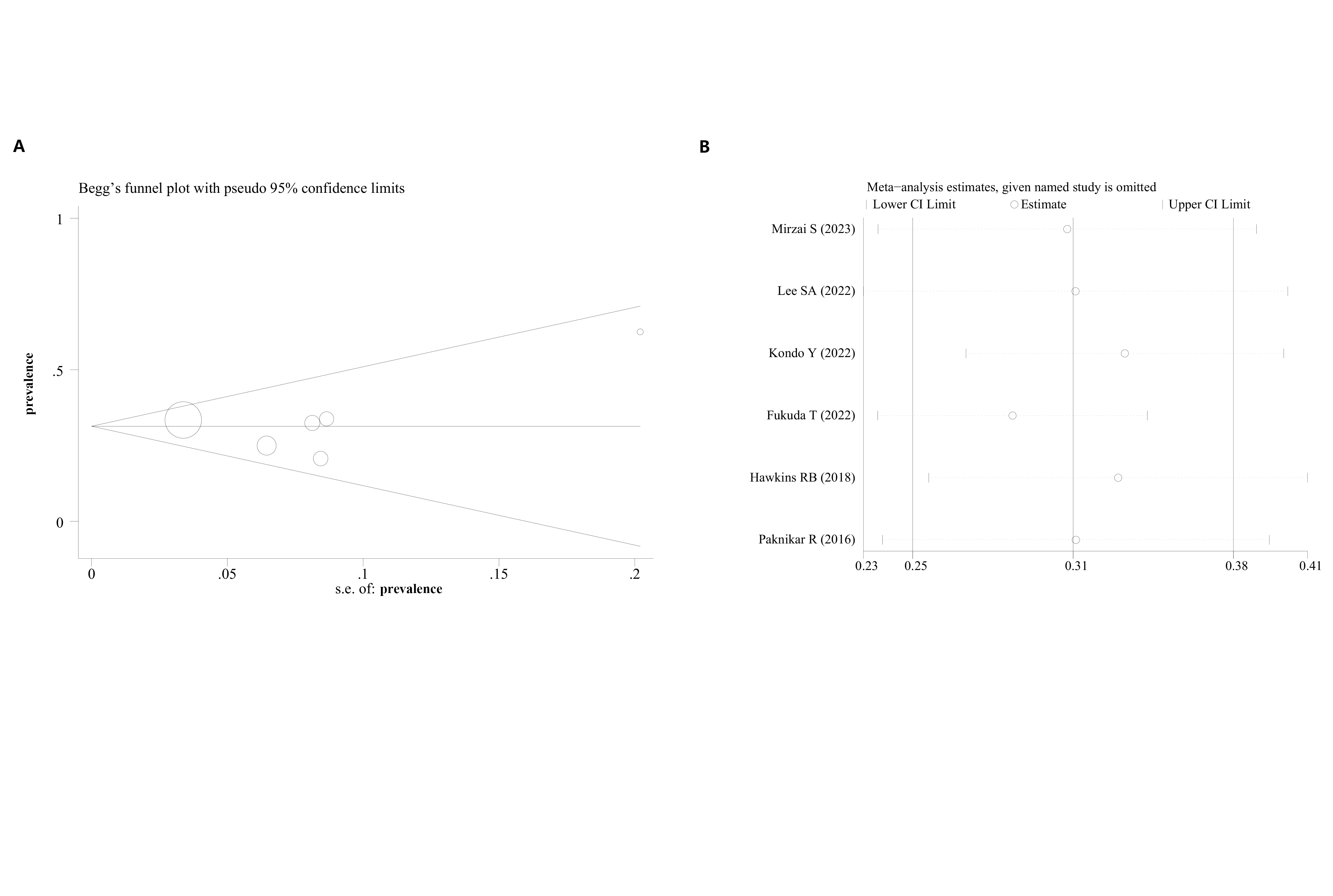

Supplement: Supplementary file 2 [file Image_1.TIF]

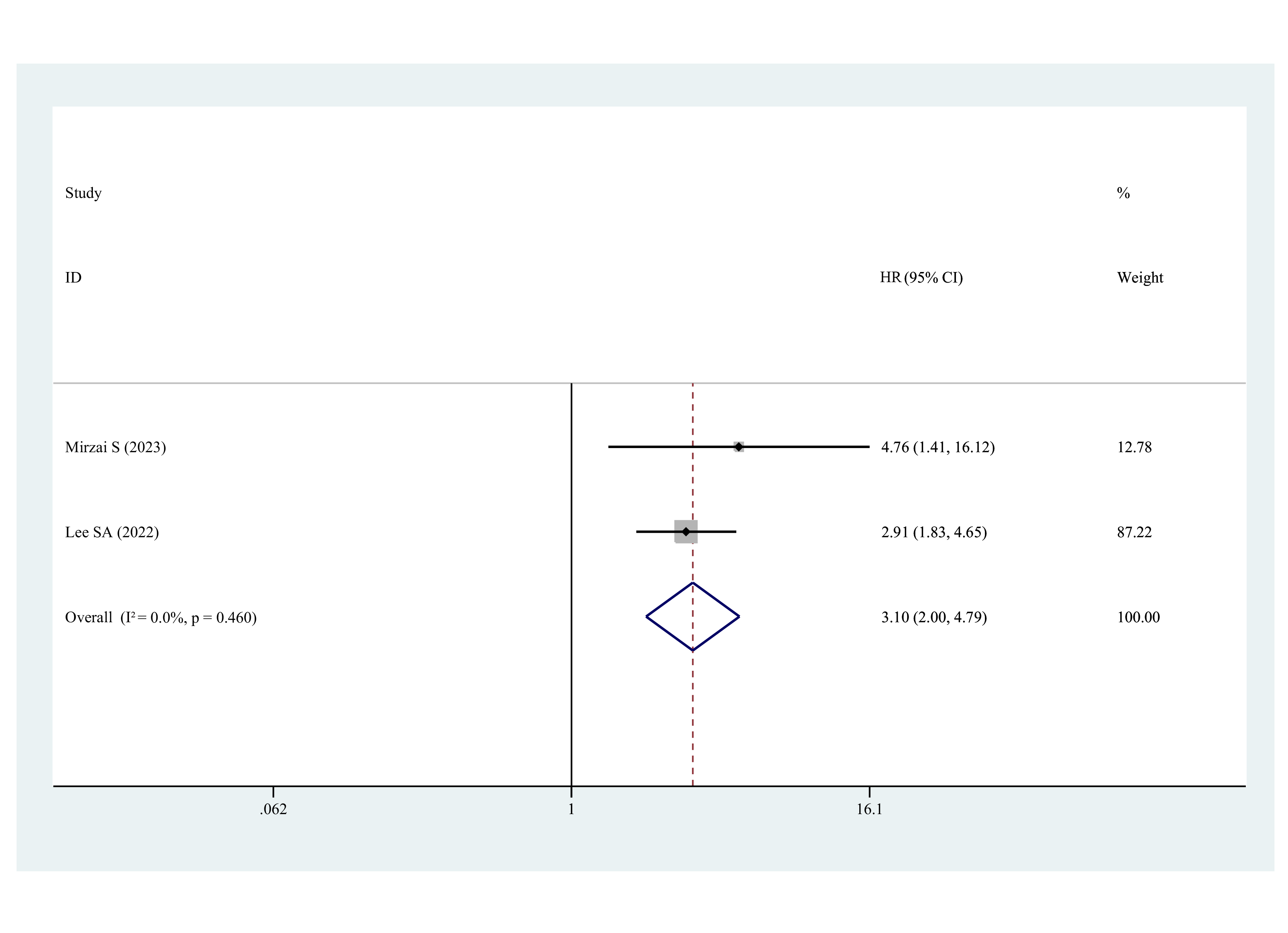

Supplement: Supplementary file 3 [file Image_2.TIF]

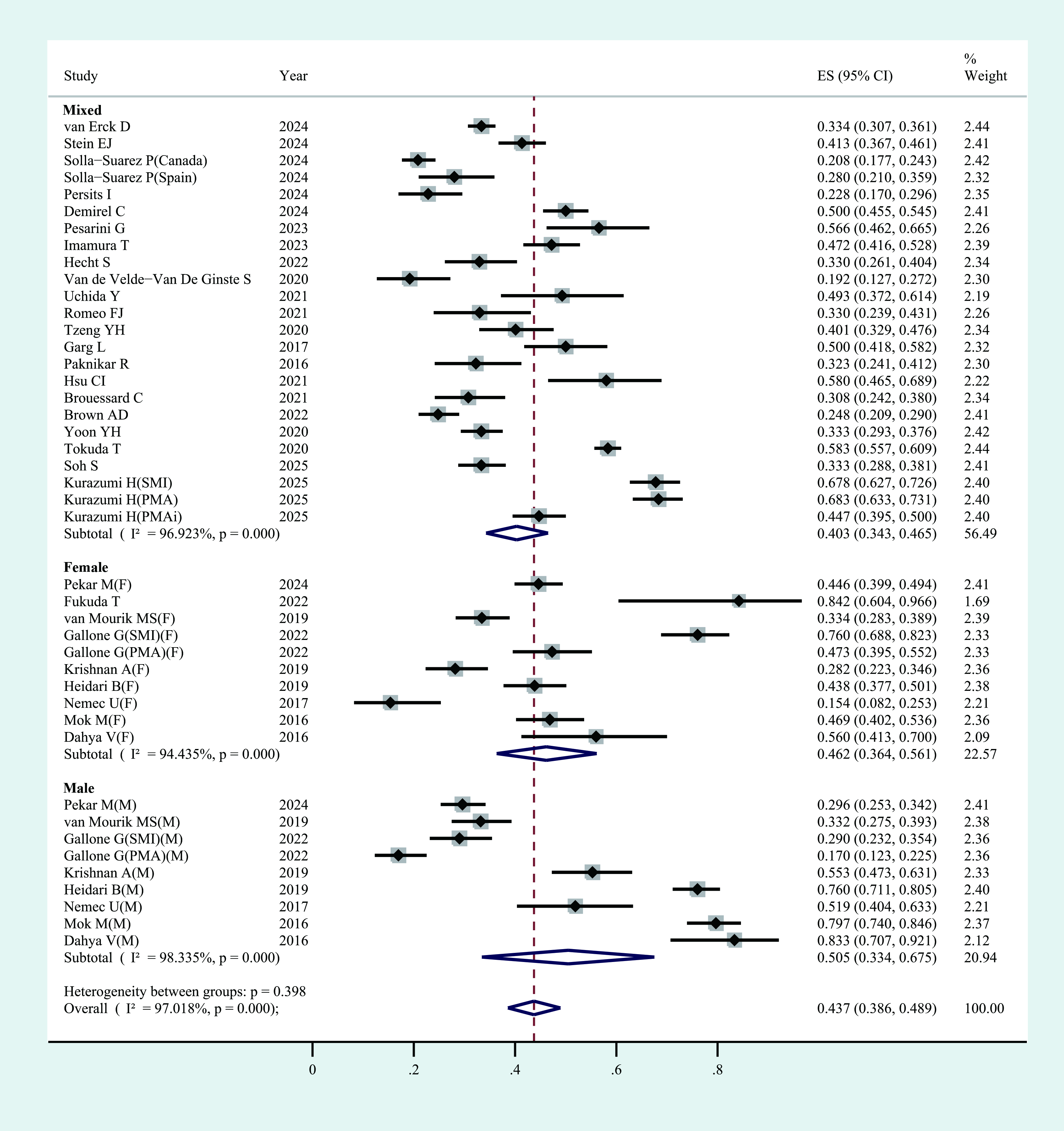

Supplement: Supplementary file 4 [file Image_3.TIF]

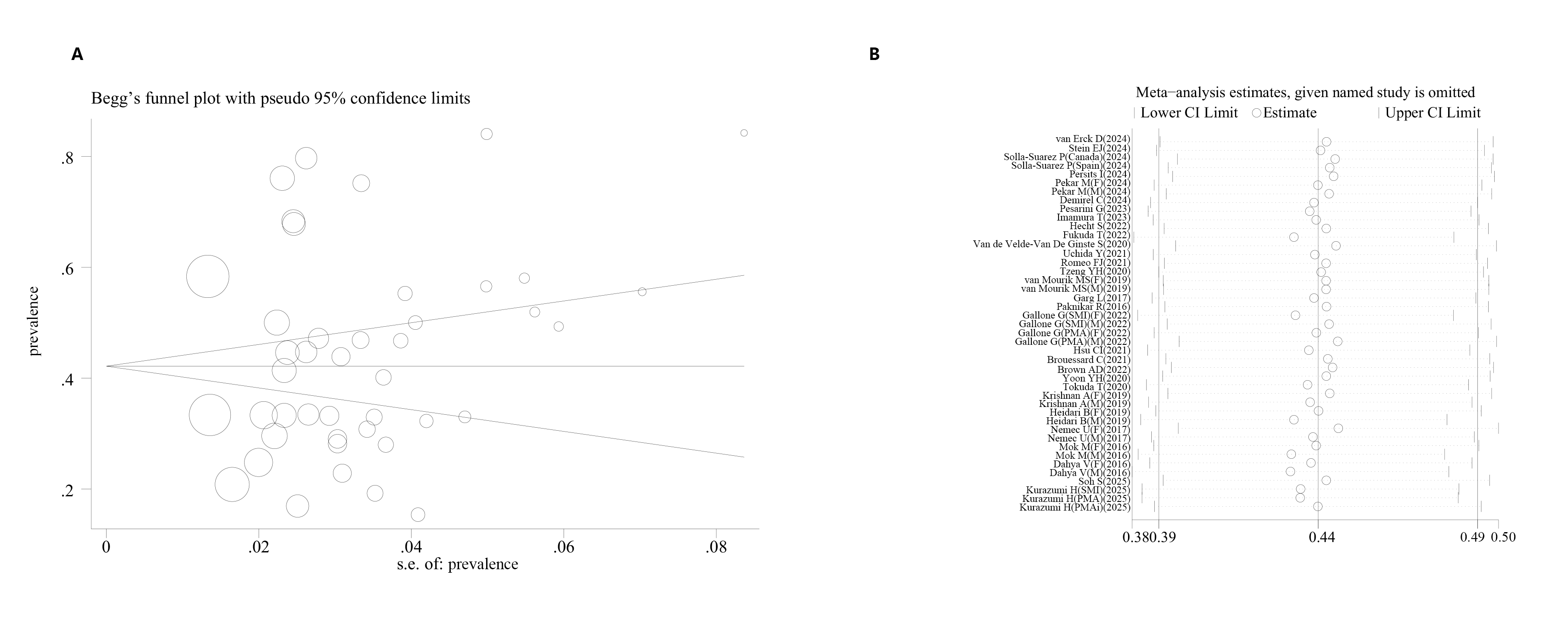

Supplement: Supplementary file 5 [file Image_4.TIF]

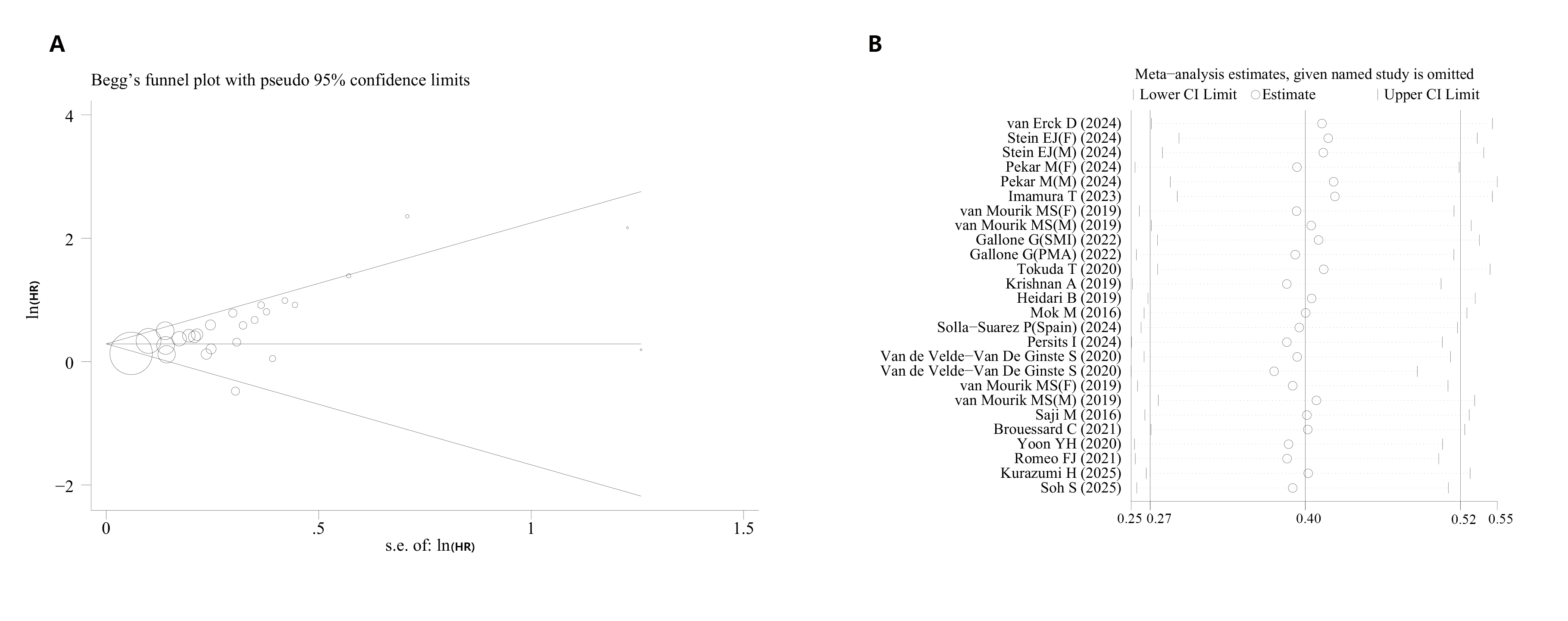

Supplement: Supplementary file 6 [file Image_5.TIF]
